# Identification of human renal cell carcinoma associated genes by suppression subtractive hybridization

**DOI:** 10.1054/bjoc.2001.2074

**Published:** 2001-09-01

**Authors:** M J J G Stassar, G Devitt, M Brosius, L Rinnab, J Prang, T Schradin, J Simon, S Petersen, A Kopp-Schneider, M Zöller

**Affiliations:** Department of Tumor Progression and Immune Defense, German Cancer Research Center, Heidelberg, 69120; Department of Urology, University Hospital, Ulm, 89075; Institute of Pathology, University Hospital Charité, Berlin, 10098; Department of Biostatistics, German Cancer Research Center, Heidelberg, 69120; Department of Applied Genetics, University of Karlsruhe, Karlsruhe, 76128, Germany

**Keywords:** human, renal cell carcinoma, gene expression

## Abstract

Renal cell carcinoma (RCC) are frequently chemo- and radiation resistant. Thus, there is a need for identifying biological features of these cells that could serve as alternative therapeutic targets. We performed suppression subtractive hybridization (SSH) on patient-matched normal renal and RCC tissue to identify variably regulated genes. 11 genes were strongly up-regulated or selectively expressed in more than one RCC tissue or cell line. Screening of filters containing cancer-related cDNAs confirmed overexpression of 3 of these genes and 3 additional genes were identified. These 14 differentially expressed genes, only 6 of which have previously been associated with RCC, are related to tumour growth/survival (EGFR, cyclin D1, insulin-like growth factor-binding protein-1 and a MLRQ sub-unit homologue of the NADH:ubiquinone oxidoreductase complex), angiogenesis (vascular endothelial growth factor, endothelial PAS domain protein-1, ceruloplasmin, angiopoietin-related protein 2) and cell adhesion/motility (protocadherin 2, cadherin 6, autotaxin, vimentin, lysyl oxidase and semaphorin G). Since some of these genes were overexpressed in 80–90% of RCC tissues, it is important to evaluate their suitability as therapeutic targets. © 2001 Cancer Research Campaign


British Journal of Cancer (2001) 85(9), 1372–1382
© 2001 Cancer Research Campaign
doi: 10.1054/ bjoc.2001.2074, available online at http://www.idealibrary.com on IDEAL

http://www.bjcancer.com

### Identification of human renal cell carcinoma associated genes by suppression subtractive hybridization

**MJJG Stassar[1,2], G Devitt[1], M Brosius[1], L Rinnab[3], J Prang[3], T Schradin[3], J Simon[3], S Petersen[4,5], A Kopp-Schneider[6] and M Zöller[1,7]**

[1]Department of Tumor Progression and Immune Defense, German Cancer Research Center, 69120 Heidelberg; [3]Department of Urology, University Hospital, 89075 Ulm; [4]Institute of Pathology, University Hospital Charité, 10098 Berlin; [6]Department of Biostatistics, German Cancer Research Center, 69120 Heidelberg; [7]Department of Applied Genetics, University of Karlsruhe, 76128 Karlsruhe, Germany

**Summary** Renal cell carcinoma (RCC) are frequently chemo- and radiation resistant. Thus, there is a need for identifying biological features of these cells that could serve as alternative therapeutic targets. We performed suppression subtractive hybridization (SSH) on patient-matched normal renal and RCC tissue to identify variably regulated genes. 11 genes were strongly up-regulated or selectively expressed in more than one RCC tissue or cell line. Screening of filters containing cancer-related cDNAs confirmed overexpression of 3 of these genes and 3 additional genes were identified. These 14 differentially expressed genes, only 6 of which have previously been associated with RCC, are related to tumour growth/survival (EGFR, cyclin D1, insulin-like growth factor-binding protein-1 and a MLRQ sub-unit homologue of the NADH:ubiquinone oxidoreductase complex), angiogenesis (vascular endothelial growth factor, endothelial PAS domain protein-1, ceruloplasmin, angiopoietin-related protein 2) and cell adhesion/motility (protocadherin 2, cadherin 6, autotaxin, vimentin, lysyl oxidase and semaphorin G). Since some of these genes were overexpressed in 80–90% of RCC tissues, it is important to evaluate their suitability as therapeutic targets. © 2001 Cancer Research Campaign

**Keywords**: human; renal cell carcinoma; gene expression

Renal cell carcinoma (RCC) accounts for approximately 3% of adult malignancies and 1.4% of cancer-related deaths (Reis and Faria, 1994). The prognosis of RCC remains poor. One third of the patients already have metastases when first consulting the hospital. Another 30–40% of patients develop metastases after surgical excision of the primary tumour (Ravaud and Debled, 1999). RCC are radioresistant (Nieder et al, 1996) and more than 80% are chemoresistant (Mickisch, 1994). Since RCC are presumed to be immunogenic, several clinical trials are exploring the efficacy of cytokines, mainly interleukin 2 (IL2) and/or interferon-α (IFNα), and the transfer of lymphokine-activated killer cells (Hofmockel et al, 1997; Bukowski, 2000; Hoffman et al, 2000). Despite these new options, the median survival time of patients with metastatic disease still remains only 6–8 months and the overall 5-year survival rate is less than 5% (Moch et al, 2000; Motzer and Russo, 2000). Thus, there is an urgent requirement for alternative therapeutic modalities.

The current strategy is to design therapeutic approaches based on specific biological features of each tumour type. These include (i) the aberrant expression of genes which can be recognized by the immune system as foreign (Pawelec et al, 1999; Wang and Rosenberg, 1999; Bremers and Parmiani, 2000); (ii) gene products related to the formation of new blood vessels (neoangiogenesis), since they are essential for tumour expansion and metastatic settlement (Harris and Thorgeirsson, 1998; Kerbel, 2000; Rosen, 2000) and (iii) the altered expression of adhesion molecules, matrix-degrading enzymes, their receptors and inhibitors, which are a further requisite of metastatic spread (Huang et al, 1997; Yu et al, 1997).

Several technique enable the identification of tumour markers. Subtractive hybridization (Lamar and Palmer, 1984; Kunkel et al, 1985; Kuang et al, 1998), differential display reverse transcription-polymerase chain reaction (DD RT-PCR; Liang and Pardee, 1992) and hybridization of cDNA microarrays (reviewed in Khan et al, 1999) are frequently used to compare the expression patterns between tumour and normal tissue. Other approaches, such as serological screening (SEREX; Sahin et al, 1995) and screening of cytotoxic T lymphocyte activity against an autologous tumour cell line (De Plaen et al, 1988), are especially focused on the identification of immunogenic tumour molecules.

We have described recently the successful use of SSH using matched RCC and normal kidney tissue (Pitzer et al, 1999). In this study, we randomly selected 16 genes, which by SSH appeared to be differentially expressed. Differential expression of 9 of these 16 genes could be verified by Northern blot analysis. 2 of the 9 genes appeared to be novel. From the remaining 7 genes, expression of 5 had been associated with the malignant phenotype. To substantiate that SSH is a suitable method for the identification of differentially expressed genes, we performed a SSH with an additional pair of normal renal and RCC tissue and compared the validity of SSH with the validity of a cDNA microarray containing 588 known human cancer-related genes using the same patient's tissues. Finally, expression of 11 genes, which differed strongly between

*Received 25 January 2001*
*Revised 18 July 2001*
*Accepted 24 July 2001*

*Correspondence to:* M Zöller

[2]Current address: Recombinant Antibody Group, German Cancer Research Center, 69120 Heidelberg, Germany, [5]Current address: Exp. Immunology Branch, NCI / NIH, 10 Center Drive, Bethesda, MD 20892 USA.

RCC and normal renal tissue, was evaluated in altogether 35 matched normal kidney and RCC tissues to obtain a hint whether these RCC-associated genes may serve as diagnostic, prognostic or therapeutic targets.

### MATERIAL AND METHODS

### Cell culture and tissue samples

Human renal cell carcinoma cell lines Caki 1, Caki 2, KTCTL-2, KTCTL-28, KTCTL-84, KTCTL-128, A-498, 769-p and 786-O were obtained from the tumour bank of the German Cancer Research Center. Lines were cultured in RPMI-medium supplemented with 10% fetal calf serum (FCS). The NSCLC lines D51, D97 and D117 have been established by one of the authors (SP). Cells were grown in Leibovitz 15 supplemented with 15% FCS and L-glutamine. The LC DMS79, H2170, H2228, H446, H526, H82, N417, SHP-77 were obtained from the American Type Culture Collection, Rockville, MD; the LC lines A427, A549, COLO 668, COLO 677, COLO 699, CPC-N, DV90 were obtained from the German Collection of Microorganisms and Cell Cultures, Braunschweig, Germany. Human small airway epithelial cells (SAEC) and human bronchial epithelial cells (HBEC) were obtained from CLONETICS and were cultured under the recommended conditions for a maximum of 10 cell divisions.

Normal kidney and kidney tumour tissue samples from RCC patients were snap-frozen in liquid nitrogen immediately after surgery and stored at –80˚C. 35 pairs of RCC/normal kidney tissue have been used.

### Isolation of RNA

Total RNA was isolated from human tumour cell lines and human tissue samples using Tri Reagent™ (Sigma, Taufkirchen, Germany) as per the manufacturer's instructions. Poly $A^+$ mRNA was isolated from total RNA samples with mini-oligo (dT) cellulose spin columns (Peqlab, Erlangen, Germany) as per the manufacturer's instructions.

### RNA gel electrophoresis and northern blot hybridization

Total RNA (20 µg) from 9 RCC cell lines and normal kidney and kidney tumour tissue from 35 different RCC patients was run for 3 to 4 hours at 80 V on a 1 × MOPS/1.2% agarose gel containing ethidium bromide and 2.2 M formaldehyde. The RNA was transferred to a positively charged nylon membrane (Hybond™-N+, version 2.0; Amerham-Pharmacia, Freiburg, Germany) by overnight capillary blotting with 20 × SSC.

For Northern blot hybridization, RNA blots were prehybridized at 42˚C for at least 1 hour and hybridized overnight with 25 ng denatured probe DNA that was labelled with 50 µCi [$^{32}$P]dCTP using Rediprime™ II (Amersham-Pharmacia). The next day the blots were washed 15 minutes in 1 × SSC/1% SDS at 42˚C, 15 minutes in 0.2 × SSC/1% SDS at 42˚C and 15 minutes in 0.2 × SSC/1% SDS at 55˚C. The blots were exposed to X-ray film (Hyperfilm™ MP, Amersham-Pharmacia). The size of the identified transcripts was determined by the position of the 18S (1.9 kb) and 28S (4.7 kb) rRNA bands. As a control for the amount of RNA loaded on the gel, blots were hybridized with glycerinaldehyde-phosphate dehydrogenase (GAPDH). For reprobing, the blots were stripped for 15 minutes in boiling 40 mM Tris-HCl (pH 7.5) with 0.1 × SSC and 1% SDS.

### Suppression subtractive hybridization (SSH)

Analysis of differentially expressed genes in human renal cell carcinoma (RCC) patient (T9) as well as of a pool of 6 RCC was performed by suppression subtractive hybridization (SSH) using the CLONTECH PCR-Select™ cDNA Subtraction Kit (Clontech, Heidelberg, Germany). In short, 2 µg of both kidney tumour and normal kidney poly $A^+$ RNA from the same RCC patient (T9) and the pooled probes, respectively, were used for double strand cDNA synthesis and the resulting cDNA was digested with Rsa I. The digested tumour cDNA was split into 2 and ligated to either adaptor 1 or adaptor 2R. For the subtraction, an excess of normal kidney cDNA was added to the adaptor-ligated kidney tumour cDNA and the samples were heat denatured and allowed to anneal. During the first hybridization the Rsa I-digested normal kidney cDNA was mixed with either adaptor 1-or 2R-ligated RCC cDNA and incubated at 68˚C for 8 h. The second hybridization, in which the 2 samples from the first hybridization were mixed together and to which freshly denatured kidney cDNA was added, was performed overnight at 68˚C. New hybrid molecules with different adaptors on each end were formed during this step and represented the differentially expressed cDNAs in RCC that were subsequently selectively amplified by 2 polymerase chain reactions (PCR): the first PCR with a primer that binds to both adaptor 1 and 2R and the second PCR with 2 nested primer that bind to adaptor 1 and 2R, respectively. The PCR products after both the first and second PCR reaction were analyzed on a 1 × TAE/2% agarose gel containing ethidium bromide. The PCR mixture, containing enriched differentially expressed transcripts, was cloned into the PCR® 2.1-TOPO (Invitrogen, Groningen, the Netherlands) and the sequence was analysed.

### Sequence analysis

Sequence analysis was performed with 3 µg of miniprep DNA using the T7 Sequenase v2.0 7-deaza-dGTP Sequencing Kit (Amersham-Pharmacia). The samples were run on a 6% polyacrylamide/8 M urea gel in 1 × TBE. The gel was dried under vacuum at 80˚C for 2 hours and exposed overnight to X-ray film (Hyperfilm™ MP, Amersham-Pharmacia).

### Vascular endothelial growth factor (VEGF) isoform analysis

The VEGF isoform pattern was analysed in T9 kidney tumour tissue and normal kidney of the same patient by reverse transcription-polymerase chain reaction (RT-PCR) as described by Tomisawa et al (1999). In short, 1 µg total RNA was reversely transcribed using 100 ng $dT_{18}$ primer and VEGF cDNA fragments were amplified by 30 cycles of PCR consisting of 1 minute at 94˚C, 1 minute at 55˚C and 2 minutes at 72˚C, using the following primers: V-S: 5′-AGCCATCCTGTGTGCCCCTGATG-3′, V-S4: 5′-GGATCAAACCTCACCAAGGCC-3′, V-A: 5′-GCGAATTC-CTCCTGCCCGGCTCAC-3′, V-A7: 5′-CTTTCTCCGCTCT-GAGCAAGGC-3′. PCR with V-S and V-A gives $VEGF_{121}$ (243 bp), $VEGF_{165}$ (375 bp), $VEGF_{189}$ (447 bp) and $VEGF_{106}$ (498 bp) fragments. PCR with V-S4 and V-A7 gives $VEGF_{165}$ (165 bp), $VEGF_{189}$ (204 bp) and $VEGF_{206}$ (255 bp) fragments.

© 2001 Cancer Research Campaign 

### Atlas cDNA expression array hybridization

2 identical Atlas-membranes (Atlas Human Cancer cDNA Expression Array, #7742-1; Clontech) were hybridized with either cDNA from normal kidney or from autologous kidney tumour following the manufacturer's protocol. The next day the membranes were washed 4 times with 2 × SSC/1% SDS for 30 minutes at 68˚C, and twice with 0.1 × SSC/0.5% SDS for 30 minutes at 68˚C.

The membranes were sealed in plastic wrap and X-ray films were exposed to the membranes overnight to 3 days at –80˚C with intensifying screens. PCR products of differentially expressed genes were used as probes in Northern blot hybridization to verify differential hybridization signals on the Atlas cDNA expression arrays.

### Hybridization of testis cDNA library arrays

High-density filter arrays containing full length cDNAs from a human testis library (Library No. 565, part 1; Experiment No. 275; Filter No. 1; Replica No. 128 and 129) were obtained from the Resource Center in the German Human Genome Project (Berlin, Germany). Blots were prehybridized for at least 1 hour at 65˚C. Probe DNA (25 ng) was labelled with 50 μCi [$^{32}$P]dCTP using Rediprime™ II (Amersham-Pharmacia) and added to the prehybridization solution after denaturation. After overnight hybridization at 65˚C the filters were washed once in 40 mM sodium phosphate/0.1% SDS at 65˚C for 20 minutes. Signals were detectable on X-ray film (Hyperfilm™ MP, Amersham-Pharmacia) after an overnight exposure at –80˚C with intensifying screens.

### Statistics

Statistical evaluation was done by the Fisher's exact test or the exact Jonckheere–Terpstra test.

### RESULTS

### Screening for differentially expressed genes in RCC by SSH

To search for new biological targets that might be useful in RCC therapy, we performed SSH with cDNA from normal kidney and tumour tissue from one patient (T9). Subtraction was performed in one direction resulting in the cloning of cDNA fragments representing genes overexpressed in T9. Over 100 clones were analysed further. First, differential expression was verified by hybridizing the clones back to Northern blots containing RNA from T9. The differential expression of 54 out of 104 clones (roughly 50%) was confirmed. 30 of these cDNA clones, despite being differentially expressed, displayed weak expression in the RCC tissue analysed (T9) and therefore were excluded from further analysis. The remaining 24 cDNA fragments, some of which are shown in Figure 1, were strongly expressed in the RCC tissue. RNA from a panel of 9 RCC tissues and matched normal kidney tissues were hybridized with these 24 clones. 3 clones (SSH-26, -33 and -42) were expressed only in T9 and were thus excluded from additional analysis. The remaining 21 clones were sequenced. Sequence analysis revealed that several of the 21 cDNA clones represented fragments from the same gene. Thus, from the original 104 clones, 11 genes were identified as being highly overexpressed in a panel of RCC tissues.

### Identification of overexpressed genes in RCC

9 of these 11 genes have been described before, i.e. sequence analysis revealed 95–100% homology at the cDNA level and corresponding sizes of the SSH-identified gene transcripts with the deposited description. 2 'newly defined' genes have meanwhile been identified. A 418 bp fragment of the 'novel' gene *SSH-28* showed 96% homology at the cDNA level and 100% homology at the protein level to the human MLRQ subunit of the NADH: ubiquinone oxidoreductase complex in mitochondria, also called the C1 respiratory complex (C1-RC) (accession No AAF80760). The second 'novel' gene, *SSH-58*, has been identified as the recently cloned human homologue of murine semaphorin G (SemG) (accession No AB040878). Expression of these genes in several kidney and RCC tissue pairs is demonstrated in Figure 2. The overall expression profiles are listed in Tables 1 and 2, which provide in addition an analysis of 9 RCC lines as well as 2 normal lung

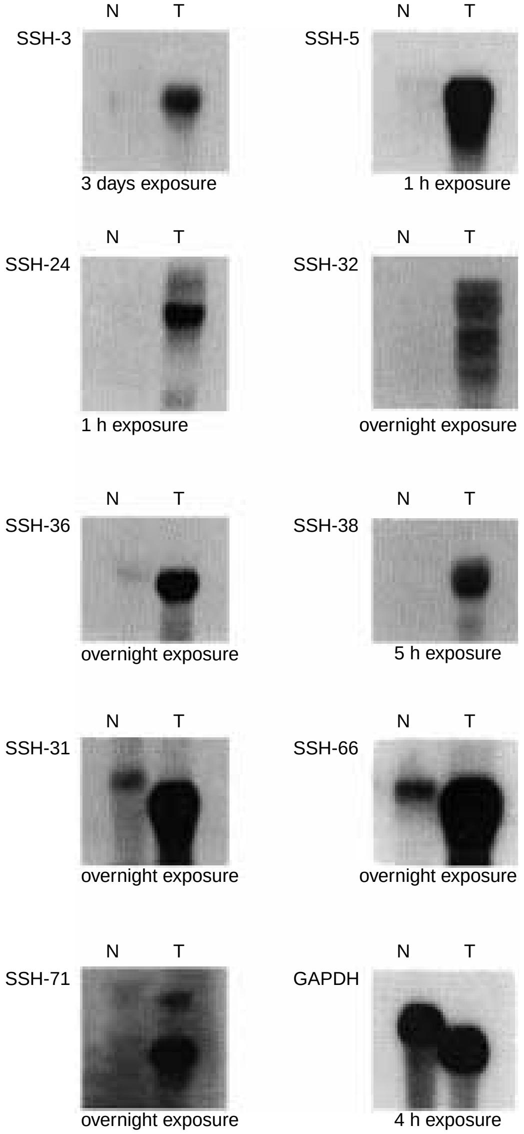

**Figure 1** Expression by Northern blot analysis of genes found by SSH. Expression in normal kidney (N) and kidney tumour tissue (T) of one RCC patient (T9). For RNA loading control, blots were hybridized with GAPDH

*© 2001 Cancer Research Campaign*

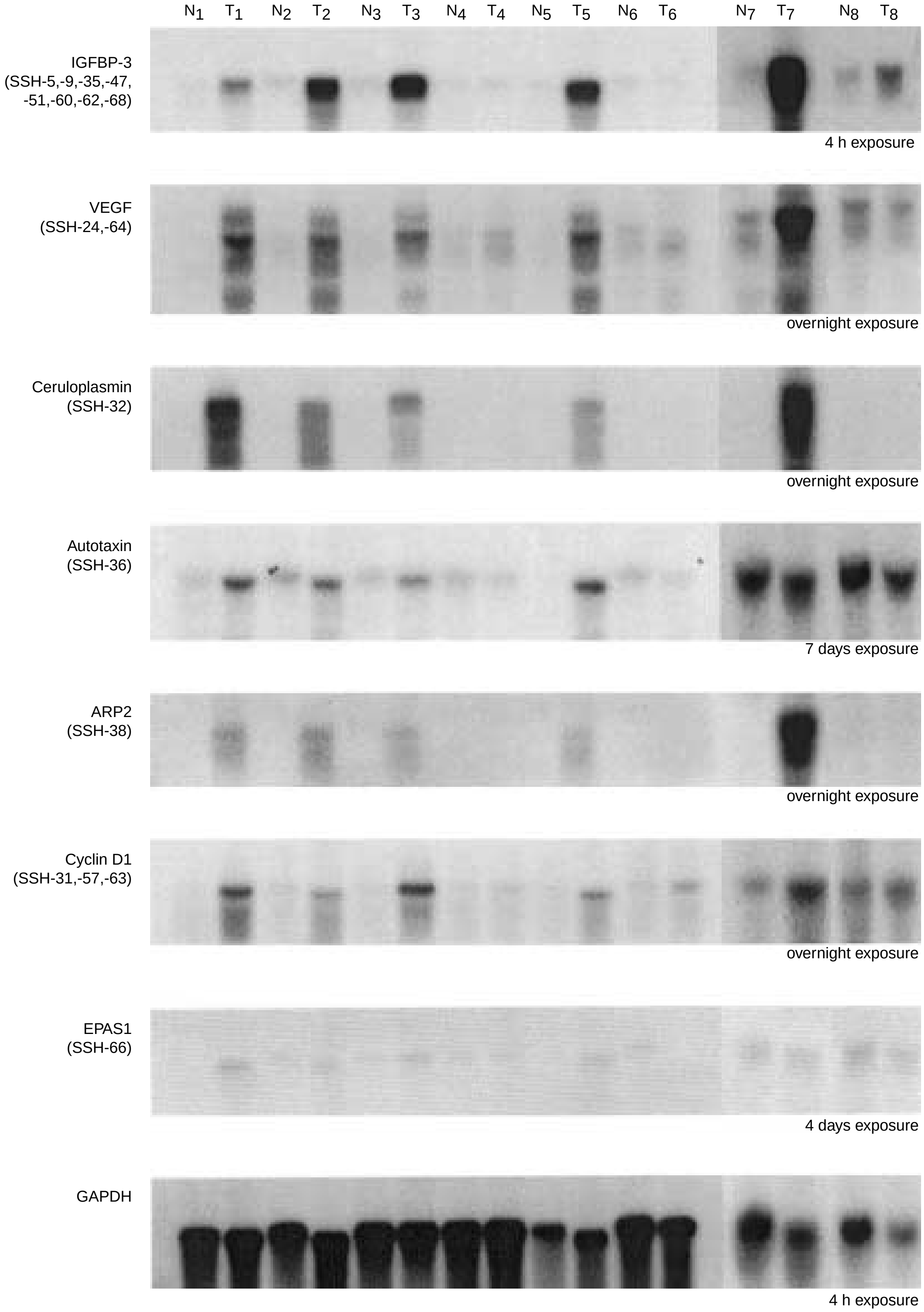

**Figure 2** Expression by Northern blot analysis of genes found by SSH. Expression in normal kidney (N) and kidney tumour tissue (T) of 8 RCC patients. For RNA loading control, blots were hybridized with GAPDH. It should be noted that in sample 8 the amount of normal kidney RNA exceeded the one of the RCC

epithelial lines, 3 non-small-cell lung carcinoma lines (NSCLC) and 15 lung carcinoma lines (LC) to obtain an impression on the selectivity of the expressed genes for RCC. Interestingly, functional activities of these 11 gene products have been associated with different stages of tumour development, namely proliferation, cell survival, neoangiogenesis and adhesion/motility.

Insulin-like growth factor-binding protein 3 (IGFBP-3) and cyclin D1 are known to be involved in cell proliferation. IGFBP-3

© 2001 Cancer Research Campaign 

**Table 1** Identification of genes differentially expressed in RCC

| RCC Tissue[b] | Differentially expressed genes[a] | | | | | | | | | | |
|---|---|---|---|---|---|---|---|---|---|---|---|
| | **IGFBP3** | **Cycl.D1** | **VEGF** | **EPAS1** | **CP** | **ARP2** | **Pcdh2** | **ATX** | **LO** | **C1-RC** | **SemG** |
| RCC T4 | – | ± | + | – | – | – | – | – | – | – | – |
| RCC T6 | – | + | ± | – | – | – | – | – | – | – | – |
| RCC T8 | +++ | + | ± | – | – | – | – | nd | – | – | – |
| RCC T5 | +++ | ++ | +++ | + | ++ | ++ | – | +++ | – | + | – |
| RCC T3 | +++ | +++ | +++ | ± | ++ | ++ | – | +++ | ± | ± | + |
| RCC T1 | ++ | +++ | +++ | + | +++ | ++ | – | +++ | + | + | ++ |
| RCC T2 | +++ | ++ | +++ | ± | +++ | ++ | – | ++ | + | ++ | ++ |
| RCC T7 | +++ | ++ | ++ | ± | +++ | +++ | – | nd | + | ++ | ++ |
| RCC T9 | +++ | +++ | +++ | ++ | +++ | +++ | + | +++ | ++ | ++ | ++ |
| RCC lines | | | | | | | | | | | |
| 769P | ± | nt | – | – | +++ | – | nt | – | – | – | – |
| 786–0 | ++ | ++ | ++ | ++ | ++ | + | – | – | – | – | – |
| A498 | +++ | ++ | ++ | ++ | +++ | + | ± | – | – | – | – |
| Caki–1 | ++ | + | nt | nt | ++ | nt | ± | – | nt | +++ | – |
| Caki–2 | +++ | + | nt | nt | + | nt | – | – | nt | +++ | – |
| KTCL–2 | ± | nt | ± | – | – | – | nt | – | – | – | – |
| KTCL–28 | +++ | + | ± | ± | ++ | – | ± | – | ++ | – | + |
| KTCL–84 | +++ | ++ | + | + | ++ | – | ± | – | ++ | – | ± |
| KTCL–128 | +++ | ++ | ± | ± | +++ | – | ± | – | +++ | – | + |

[a]the degree of overexpression is indicated by +++: very strong, ++: strong, +: distinct, ±: weak, but differential; nd (not differential) indicates expression in normal kidney tissue, nt: not tested.
[b]RCC tissue were grouped according to expression profiles, RCC T9: RCC tissue of the same patient that was used for SSH.

**Table 2** Expression of RCC-associated genes in normal lung and lung carcinoma

| Lung Tissue[a] | Differentially expressed genes | | | | | | | | | | |
|---|---|---|---|---|---|---|---|---|---|---|---|
| | **IGFBP3** | **Cycl.D1** | **VEGF** | **EPAS-1** | **CP** | **ARP2** | **Pcdh2** | **ATX** | **LO** | **C1-RC** | **SemG** |
| SAEC (1)[b] | 0 | 0 | 1 | 0 | 0 | 1 | 0 | 0 | 0 | 1 | 0 |
| HBEC (1)[b] | 0 | 0 | 1 | 0 | 0 | 1 | 0 | 0 | 0 | 0 | 0 |
| NSCLC (3)[b] | 1 | 0 | 3 | 0 | 0 | 3 | 0 | 0 | 0 | 0 | 0 |
| LC (15)[b] | 2 | 0 | 15 | 0 | 0 | 2 | 0 | 0 | 0 | 2 | 0 |

[a]SAEC: small airway epithelial cells, HEBC: human bronchiolar epithelial cells, NSCLC: non-small cell lung carcinoma lines, LC: lung carcinoma lines;
[b]in brackets: number of samples.

was overexpressed in 7/9 RCC tissues and all 9 RCC lines. It also was expressed in 3 lung carcinoma lines. Cyclin D1 was overexpressed in 9/9 RCC tissue and all tested RCC lines, while in none of the 18 lung carcinoma lines cyclin D1 was detected.

4 of the 11 differentially expressed genes encode proteins involved in angiogenesis: vascular endothelial growth factor (VEGF), endothelial PAS domain protein 1 (EPAS1), ceruloplasmin (CP) and angiopoietin-related protein 2 (ARP2). Overexpression of VEGF could be demonstrated in all RCC tissues, 6 out of 7 RCC lines and in all lung-derived cell lines, i.e. in normal lung epithelial cell lines as well as lung carcinoma lines. Different VEGF isoforms, encoding polypeptides consisting of 121, 145, 165, 189 and 203 amino acids have been described (Neufeld et al, 1999). To test which of the isoforms were overexpressed in the original kidney tumour (T9), an RT-PCR was performed with primers that lead to distinct bands for the different isoforms (Tomisawa et al, 1999). PCR with primers V-S and V-A gives $VEGF_{121}$ (243 bp), $VEGF_{165}$ (375 bp), $VEGF_{189}$ (447 bp) and $VEGF_{106}$ (498 bp) fragments. PCR with the primers V-S4 and V-A7 gives $VEGF_{165}$ (165 bp), $VEGF_{189}$ (204 bp) and $VEGF_{206}$ (255 bp) fragments. GAPDH primers were used as a control. All VEGF isoforms were overexpressed in kidney tumour T9 (Figure 3).

Differential expression of EPAS1 was seen in 6 out of 9 RCC tissues. It was expressed in 5 out of 7 RCC lines, but not in any of the lung samples. CP was distinctly expressed in 6 out of 9 RCC tissues and all except one RCC line. It was not detected in lung carcinoma lines. ARP2 was expressed in 6 RCC tissues, but only in 2 out of 7 RCC lines. Both a bronchial epithelial and a small airway epithelial cell line were ARP2 positive. Also, 3/3 NSCLC, but only 2/15 LC were ARP2 positive.

With respect to adhesion-related molecules, we recovered 3 genes: protocadherin 2 (Pcdh2; also called cadherin-like 2), autotaxin (ATX) and lysyl oxidase (LO). Protocadherin 2 was not detected in the other 8 RCC tissues, but in 5 out of 7 RCC lines, albeit weakly. No signal was seen in Northern blots of lung carcinoma lines. ATX, also called autocrine motility factor, was clearly overexpressed in 5 RCC tissues. In 2 samples, ATX was also strongly expressed in normal kidney tissue. Neither the RCC lines nor the lung carcinoma lines expressed ATX. Expression of lysyl oxidase (data not shown) was upregulated in 5 RCC tissues and was not detectable in lung carcinoma lines, but in 3 out of 7 RCC lines.

The 'novel' gene *C1-RC* was overexpressed in 6 RCC tissues, 2 out of 9 RCC lines, the small airway epithelial cell line and 2 LC

*© 2001 Cancer Research Campaign*

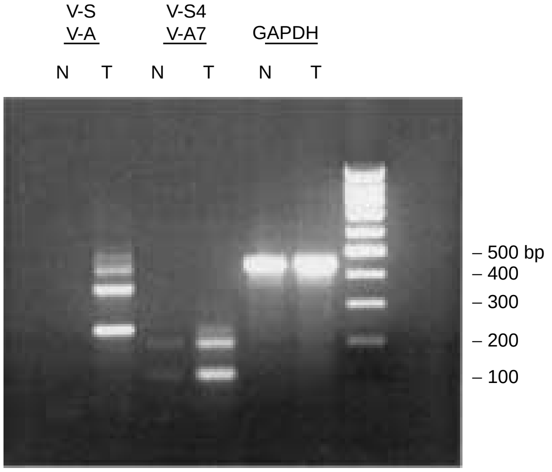

**Figure 3** Identification of vascular endothelial growth factor (VEGF) isoforms. RT-PCR of normal (N) and tumour (T) tissue of one RCC patient (T9). PCR with V-S and V-A gives VEGF$_{121}$ (243bp), VEGF$_{165}$ (375bp), VEGF$_{189}$ (447bp) and VEGF$_{106}$ (498bp) fragments. PCR with V-S4 and V-A7 gives VEGF$_{165}$ (165bp), VEGF$_{189}$ (204bp) and VEGF$_{206}$ (255bp) fragments. PCR with GAPDH primers served as positive control lines. The second ‘novel’ gene, *SemG*, was differentially expressed in 5 RCC tissues and 3 RCC lines. Lung carcinoma lines did not express *SemG*.

Taken together, from 14 genes differentially expressed in the tumour and normal kidney tissue of one patient, overexpression of 11 genes was also detected in additional RCC tissue samples and/or RCC lines. Interestingly, with the only exception of IGFBP3, none of these genes were differentially expressed in lung cancer versus normal lung tissue lines.

### Controlling reliability of SSH

To confirm our findings by SSH of one matched pair of normal kidney tissue versus a RCC, SSH was repeated using a pool of 6 RCC versus the matched pool of normal kidney tissue. Northern blotting revealed that 8 genes were differentially expressed in the pool of RCC and in additional RCC as compared to matched normal kidney tissue. Interestingly, 5 of these 8 genes had also been recovered by the T9 tumour, i.e. *IGFBP-3*, *VEGF*, *ARP2*, *CP* and *cyclin D1*. There were 2 additional genes, which were strongly upregulated in RCC tissue, *BACE2* (beta site amyloid precursor protein cleaving enzyme) and *SUPT5H* (human homologue of the suppressor of transposon Ty). Expression levels of a third gene, *ARPP-19*, were found to vary widely in normal kidney tissue. Therefore, differential expression of this gene has not been further pursued.

Finally and to obtain a more comprehensive view of genes differentially expressed in RCC, the normal kidney and T9 RCC tissue used for SSH were tested on 2 dot blots (Atlas blots) which contain 588 known human cancer-related genes (Figure 4). Putatively differentially expressed genes were tested on Northern blots to eliminate false positives. 3 of the genes detected by SSH, namely *cyclin D1*, *VEGF* and *IGFBP-3*, were also recovered by Atlas blot hybridization. Of the remaining 10 genes, only *cadherin 6*, *vimentin* and *epidermal growth factor receptor* (*EGFR*) could be verified by Northern blot analysis. None of the genes seemingly overexpressed in normal kidney tissue could be verified by Northern blot analysis (data not shown).

In an attempt to isolate potentially immunogenic antigens, particularly so called cancer-testis antigens, 2 identical filters containing full-length cDNAs of human testis were hybridized with normal kidney cDNA and the corresponding subtracted T9 RCC cDNA. Unfortunately, from 25 seemingly differentially expressed clones, none could be verified after rescreening by Northern blot hybridization (data not shown).

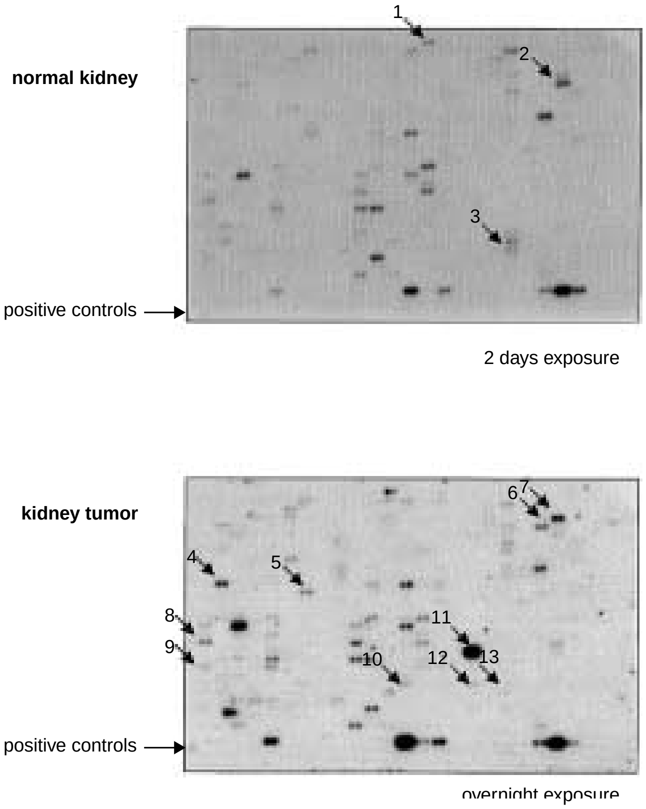

**Figure 4** Hybridization of Atlas blots with normal kidney and RCC cDNA. Differentially expressed genes are marked by arrows. The apparently overexpressed genes in normal kidney tissue c-fos (1), IGFBP-5 (2) and hepatocyte growth factor (3) could not be verified in Northern blot analysis; From the genes overexpressed in RCC tissue cyclin D1 (4), vimentin (5), EGFR precursor (6), IGFBP-3 (7), cadherin 6 (10) and VEGF (11) were verified by Northern blot analysis, but not CD59 (8), collagen type 1 (9), BMP3 (12) and early growth response protein (13). The last row of each blot contains positive controls

### Expression profile and clinical features

As shown in Figure 2, there were clear differences in the expression profiles between the 9 RCC samples tested. 5 RCC tissues displayed rather uniform expression profiles of the described differentially expressed genes, while expression profiles of 4 RCC tissues differed, with expression of only 2, 3 and 8 of the 11 genes, respectively. Thus, it became tempting to speculate that different gene expression profiles might correlate with histology, grading or staging of the tumour. To support the assumption, Northern blots were performed with an additional 26 matched normal renal and RCC tissues Blots were hybridized with probes of *IGFBP3*, *Cyclin D1*, *C1-RC*, *VEGF*, *CP*, *ARP2*, *ATX*, *LO*, *SemG* and with probes of *BACE2* and *SUPT5H*, two genes identified by using a pool of normal kidney tissue and matched RCC for SSH.

Table 3 shows the reactivity profiles when samples were grouped according to histology, grading and TNM staging. The individual RCC-reactivity profiles, grouped according to the histological type are, in addition, shown in Figure 5. Although with the

**Table 3** Correlation between differential gene expression, histology, grading and staging

| Clinical features | IGFBP3 | Cycl.D1 | C1-RC | SUPT5H | VEGF | CP | ARP2 | ATX | LO | SemG | BACE2 |
|---|---|---|---|---|---|---|---|---|---|---|---|
| Histology | | | | | | | | | | | |
| Clear (20) | 17/20 | 12/18 | 16/18 | 15/20 | 15/20 | 11/18 | 14/18 | 8/20 | 9/20 | 8/16 | 3/13 |
| *P* value (vs all other types)[1] | ns[2] | ns | <0.0001 | 0.007 | 0.02 | ns | 0.005 | 0.02 | 0.01 | 0.04 | 0.002 |
| Clear and granular (3) | 3/3 | 2/2 | 0/3 | 0/3 | 0/3 | 0/2 | 0/2 | 0/3 | 0/3 | 0/3 | 3/3 |
| *P* values (vs clear)[1] | ns | | 0.007 | 0.03 | 0.03 | | | ns | ns | ns | 0.02 |
| Granular (3) | 0/3 | 0/3 | 0/3 | 0/3 | 0/3 | 0/3 | 1/3 | 0/3 | 0/3 | 0/3 | 1/2 |
| *P* value (vs clear)[1] | 0.01 | ns | 0.007 | 0.03 | 0.03 | ns | ns | ns | ns | ns | |
| Oxyphil (3) | 2/3 | 2/3 | 0/3 | 2/3 | 3/3 | 1/3 | 0/3 | 0/3 | 0/3 | 0/3 | 3/3 |
| *P* value (vs clear)[1] | ns | ns | 0.007 | ns | ns | ns | 0.03 | ns | ns | ns | 0.02 |
| Tubulopapillary (1) | 1/1 | 0/1 | 0/1 | 0/1 | 0/1 | 1/1 | 1/1 | 0/1 | 0/1 | 1/1 | |
| Grading[3] | | | | | | | | | | | |
| GI (13) | 9/13 | 6/10 | 5/11 | 7/13 | 9/13 | 4/10 | 6/10 | 1/13 | 3/13 | 2/10 | 7/12 |
| GII (13) | 11/13 | 8/13 | 8/13 | 7/13 | 6/13 | 7/13 | 7/13 | 4/13 | 4/13 | 5/12 | 2/8 |
| GIII (5) | 3/5 | 2/5 | 3/5 | 3/5 | 3/5 | 2/5 | 3/5 | 3/5 | 2/5 | 2/5 | 1/1 |
| Staging | | | | | | | | | | | |
| T1,N0,M0 (17) | 12/17 | 8/14 | 7/15 | 8/17 | 10/17 | 5/14 | 8/14 | 1/17 | 3/17 | 5/14 | 7/12 |
| T2,N0,M0 (6) | 4/6 | 3/6 | 2/6 | 3/6 | 2/6 | 2/6 | 2/6 | 1/6 | 1/6 | 1/6 | 2/3 |
| T3a/3b,N0,M0 (5) | 4/5 | 3/5 | 4/5 | 3/5 | 3/5 | 3/5 | 3/5 | 3/5 | 2/5 | 1/4 | 0/4 |
| T3a/T4,N2,M0 (3) | 3/3 | 2/3 | 3/3 | 3/3 | 3/3 | 3/3 | 3/3 | 3/3 | 3/3 | 2/3 | 1/2 |
| *P*-value[4] | ns | ns | ns | ns | ns | ns | ns | 0.002 | 0.03 | ns | ns |

[1]*P* values are derived from Fisher's exact test, clear cell RCC were compared against all non-clear-cell RCC, the other groups were compared against clear-cell RCC as far as the minimal number of 3 samples had been tested.
[2]ns: not significant.
[3]no statistically significant differences were found in dependence of tumour grading.
[4]*P* values are derived from the exact Jonckheere–Terpsta test, which describes a trend from T1, N0,M0 towards T3/T4,N2,M0.

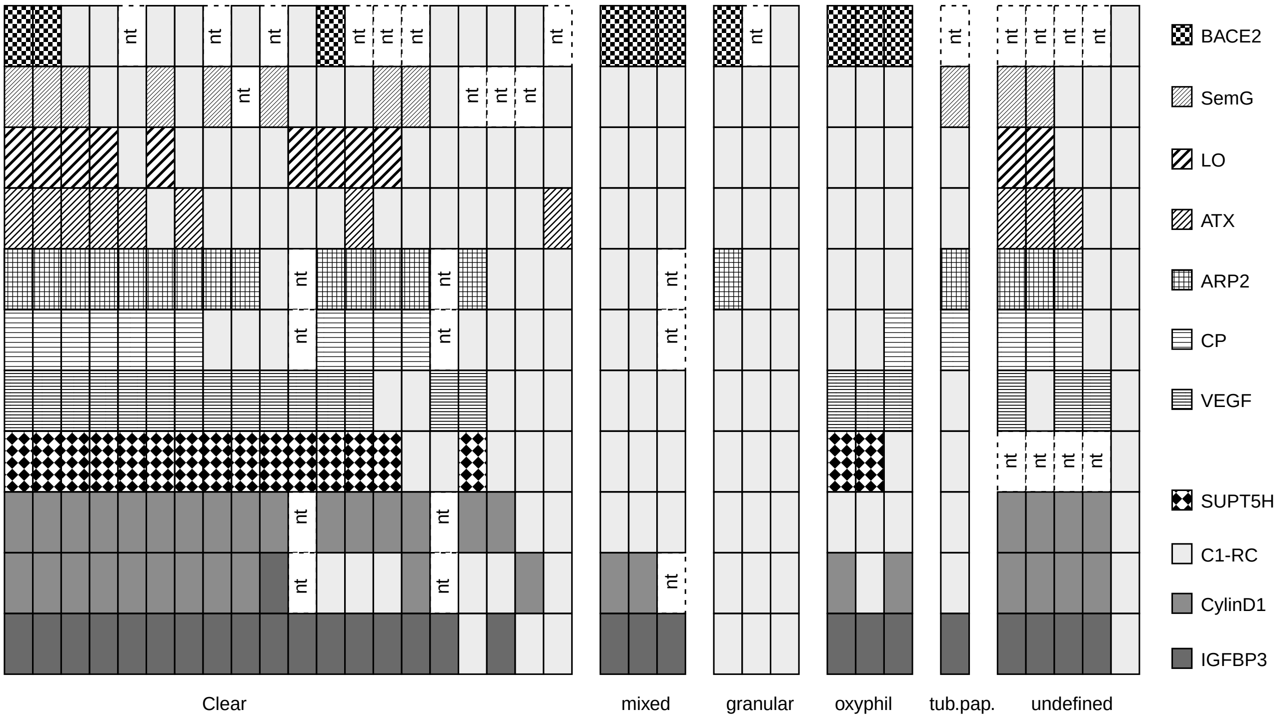

**Figure 5** Differential gene expression in histopathological subtypes of RCC: Differential expression of 11 genes was evaluated by Northern blot analysis of matched normal kidney and RCC tissue and is shown for 20 clear cell, 3 mixed clear and granular, 3 granular, 3 oxyphil, 1 tubulopapillary and 5 histologically undefined RCC. White squares indicate that the gene was not differentially expressed; nt: not tested exception of clear cell RCC, the numbers of samples of the different histological types were too small to allow a statistical analysis between these groups, they could be compared to the group of clear-cell RCC. The group of clear-cell RCC was compared to all non-clear-cell RCC. Overexpression of some of the RCC-associated genes appears to be preferentially associated with the histological type, e.g. overexpression of *C1-CR*, *SUPT5H*, *VEGF*, *ARP2*, *ATX*, *LO* and *SemG* has been mainly seen

 *© 2001 Cancer Research Campaign*

in clear cell RCC, whereas *BACE2* was overexpressed in mixed (clear and granular cell) and oxyphil RCC. The failure to detect overexpression of most of the genes in RCC of the granular type was somehow unexpected. Thus, it is desirable to confirm these expression profiles with larger numbers of samples.

We did not detect any significant differences in the gene expression profile between well versus poorly differentiated RCC. However, there has been a trend towards overexpression of *ATX* and *LO* with tumour progression. It remains to be explored in a follow-up study and evaluating 5 years survival rate, recurrence and metastatic spread, whether this trend will be of prognostic relevance.

### DISCUSSION

Until now there has been a lack of knowledge regarding renal cell carcinoma (RCC)-associated molecules that might be suitable as therapeutic targets. Our search for such molecules involved 2 different approaches. Patient-matched cDNA from normal kidney and RCC tissue were used for suppression subtractive hybridization and screening of cDNA (Atlas) arrays. 17 genes were found to be significantly overexpressed in one RCC tissue, 14 of which were also detected in additional RCC tissues and lines, but not in normal kidney tissue.

Taking into account that some of the clones identified by SSH contained fragments of identical genes, we recovered 44 differentially expressed genes, while only 6 genes were recovered by hybridization of a blot containing 588 genes known to be tumour-related. The fact that SSH and cDNA array analysis both showed a false positive rate of approximately 50% illustrates the point that all of the techniques used to identify differentially regulated genes must be confirmed by Northern blot hybridization to the original samples. The same RCC tissue (T9) was analysed by differential display RT-PCR in a previous study (Stassar et al, 1999). As compared to differential display RT-PCR, SSH yielded a higher number of differentially expressed genes and the rate of false positives was significantly lower. Thus, searching for therapeutic targets, SSH may be the more suitable method as compared to differential display RT-PCR and appears to be, at least, equal to cDNA arrays and distinct from cDNA arrays of known genes, SSH has the additional advantage of uncovering unidentified genes. By using a pool of 6 RCC versus the pool of matched normal kidney tissue for SSH, differential expression of 5 of the 11 genes identified with T9 was confirmed, which strengthens the reliability of SSH. On the other hand and without question, the screening process can be strongly accelerated by the use of cDNA arrays.

The gene products identified in this study by both methods, SSH and cDNA arrays, are involved in proliferation/resistance towards apoptosis, neo-angiogenesis and cell adhesion/motility. These features and our point of view should be discussed in some detail.

3 of the 5 tumour growth-related genes are well known. Thus, the *EGFR* gene, which has been detected by Atlas blot hybridization, is one of the key molecules in epithelial tumour cell growth regulation (Ullrich and Schlessinger, 1990). The *EGFR* might also contribute to tumour cell motility and has been associated with metastatic spread (Lager et al, 1994; Yoshida et al, 1994). Up-regulation of *EGFR* expression has frequently been found in RCC (Freeman et al, 1989; Sargent et al, 1989; Ishikawa et al, 1990).

*Cyclin D1*, named according to its cell cycle (G1 phase)-dependent appearance, sequentially activates cyclin-dependent kinases, which phosphorylate various substrates, the retinoblastoma protein being the most prominent target (Weinberg, 1995). *Cyclin D1* overexpression has been reported in many tumours including breast carcinoma and squamous cell carcinoma of the head and neck, oesophagus and cervix (Somers and Schechter, 1992; Bartkova et al, 1994; Jares et al, 1994; Zhang et al, 1994; Kurzrock et al, 1995). 2 studies reported an overexpression of *cyclin D1* in approximately 50% of RCC. In both studies no correlation with tumour stage, differentiation and survival time was found (Lin et al, 1998; Hedberg et al, 1999).

*SUPT5H* together with *SUPT4H* and *SUPT6H* are believed to play a critical role in transcription, being involved in transcription elongation and in activation of transcription (Wen and Shatkin, 1999, Kaplan et al, 2000; Yamaguchi et al, 2001). A direct involvement of *SUPT5H* in tumour progression has not yet been reported.

*IGFBP*s are usually known for their growth inhibitory effects by competitively binding insulin-like growth factor (Li et al, 1997). IGFBP-3, as well as several other *IGFBP*s, can also promote proliferation by a regulated release of insulin-like growth factor, which protects its receptor from down-regulation by exposure to high concentrations of insulin-like growth factor (Conover and Powell, 1991). One of the recently identified genes, which was selectively expressed in RCC, is a homologue of a human MLRQ subunit of the NADH: ubiquinone oxidoreductase, known as the first and largest enzyme of the mitochondrial respiratory chain (Weiss et al, 1991). Though additional experiments are required for defining the function of this subunit, we hypothesize that this molecule is responding to the high metabolic demand of tumour cells.

An emerging tumour initiates its own blood supply by stimulating surrounding vessels to grow into the tumour mass. These newly formed vessels are highly irregular and tortuous which is accompanied by hypoxia (Vaupel, 1997; Brown, 1999). Hypoxia initiates a genetic programme leading to up-regulation of key pro-angiogenic molecules and of factors directly influencing tumour cell survival (Avantaggiati, 2000). *VEGF* is known to play a crucial role in these events: it is a major inducer of neovascularization (Gerwins et al, 2000), and additionally up-regulates the expression of anti-apoptotic factors like XIAP and survivin (Tran et al, 1999). A correlation between the expression of *VEGF* and its receptor and the degree of vascularization has been described in many tumour systems including RCC (Tomisawa et al, 1999). It is also of prognostic relevance with respect to the risk of metastasis formation (Weidner, 1995). Although expression of different VEGF isoforms is frequently observed in tumour tissues, it has been described that particularly expression of the higher molecular weight isoforms $VEGF_{165}$ and $VEGF_{189}$ correlates with high vessel counts and poor prognosis (Oshika et al, 1998; Lee et al, 1999; Tomisawa et al, 1999).

Transcription of *EPAS1*, also called hypoxia-inducible factor (HIF)-2α, is induced by hypoxia. *EPAS1* is a transcription factor that binds to HIF-1 and activates downstream genes such as *VEGF* and *endothelial cell specific receptor tyrosine kinases* (Tian et al, 1997; Wiesener et al, 1997; Conrad et al, 1999). In fact, we observed a good correlation between the expression of *EPAS1* and the overexpression of VEGF (Maemura et al, 1999).

Although a direct association of angiopoietin-related proteins with tumour growth has not been reported, both *ARP2* and *CP* are indirectly involved in angiogenesis. *ARP2* acts as an anti-apoptotic factor on vascular endothelial cells (Kim et al, 1999). *CP* is responsible for the accumulation of copper ions at the apical growth cone of newly forming blood vessels (Raju et al, 1982). CP overexpression has already been described in human tumours (Kanapuli et al, 1987).

*© 2001 Cancer Research Campaign*

We also identified several genes associated with cell motility, another important factor in the process of tumour progression. *ATX* is an autocrine tumour cell motility factor (Stracke et al, 1997), whose expression also has been described to inversely correlate with cell differentiation (Yang et al, 1999). *ATX* was found to be overexpressed in a variety of tumours such as malignant melanoma (Stracke et al, 1992), teratocarcinoma (Lee et al, 1996), neuroblastoma (Kawagoe et al, 1997) and non-small-cell lung carcinoma (Yang et al, 1999). However, this is the first report of ATX overexpression in RCC. Interestingly, ATX was neither expressed in RCC lines nor in LC lines. Lysyl oxidase has been reported to influence tumour cell motility/invasiveness (Kirschmann et al, 1999) by reshaping the collagen matrix (Williamson et al, 1985). However, details on how LO functions remain to be explored.

*Protocadherin 2* has also been shown to promote metastasis formation by supporting cell adhesion (Obata et al, 1995). Overexpression of other members of the cadherin superfamily, such as *cadherin 6*, have been described in several tumours including RCC (Shimoyama et al, 1995), and the aberrant expression of *cadherin-6* correlates with poor prognosis (Paul et al, 1997; Shimazui et al, 2000). *Vimentin*, found in this study by Atlas blot hybridization and reported before by Moch et al (1999) to be aberrantly expressed in RCC, has been repeatedly shown to correlate with high metastatic potential (Thompson et al, 1994; Hendrix et al, 1996).

*BACE2* is a transmembrane aspartic protease (Bennett et al, 2000), which so far is mainly known for its involvement in Alzheimer's disease (Vassar et al, 1999). It has, however, been described that *BACE2* is differentially expressed in breast cancer cell lines and it was suggested to contribute to the proteolytic cascade in neoplastic cells, which facilitates the process of tumour progression (Xin et al, 2000). Interestingly the target molecule of *BACE2*, the amyloid beta protein precursor, has been described to be involved in the growth of human colon carcinoma cells. The authors suggest that this is due to a Kunitz-type inhibitor domain of the molecule (Meng et al, 2001). It remains to be explored whether up-regulation of the *BACE2* aspartic protease in RCC can counteract the serine protease inhibition by amyloid beta protein precursor.

The functional activity of the human homologue of *semaphorin G* in RCC also remains to be explored. Several semaphorins have been reported to be expressed in association with tumours. Overexpression of *semaphorin E* and *H* has been reported in metastases (Christensen et al, 1998; Martin-Satue and Blanco, 1999) while it has been hypothesized that *semaphorin 3f* might be involved in cell adhesion and motility (Brambilla et al, 2000). Since semaphorins are phylogenetically conserved proteins that mediate repulsive guidance events during neuronal development (Mark et al, 1997), we speculate that *SemG* will serve a similar motility function.

Three additional aspects should be mentioned. First, it is frequently argued that long-cultured tumour cell lines cannot be considered as relevant with respect to the gene expression profiles seen in primary tumours. With the exception of ATX, this does not seem to be true for RCC. On the other hand, we observed marked differences between expression profiles in RCC lines and lung cancer cell lines. This finding was also surprising since all of the genes overexpressed in RCC have been discussed to be associated in general with the malignant phenotype. Additional studies with a variety of tumour types are required to ascertain whether tumours arising from different tissues express distinct cancer-related gene profiles. It also remains to be explored, whether such expression patterns may relate to differences in the preferential target organ of metastasis. Such features of gene expression profiles could become of diagnostic relevance.

Second, one could argue that our study provides evidence for clusters of gene expression. Thus, in a first screening of 9 RCC, all or nearly all of the genes discovered by SSH were overexpressed in 5 RCC tissues, while four other RCC tissues expressed only some, but not necessarily the same genes. To corroborate the hypothesis, we tested a larger panel of kidney and RCC tissue for differentially expressed genes and compared the gene expression profile in RCC tissue with histopathology and clinical staging. Although we could define a correlation between clinical features and clusters of overexpressed genes, some of the genes, like e.g. *C1-RC* were preferentially overexpressed in clear cell RCC, whereas overexpression of *BACE2* was rare in clear cell RCC, but frequent in oxyphil RCC. We noted no correlation to the tumour grading, i.e. the expression profile appeared to be independent of whether the tumour was highly or poorly differentiated. Because with few exceptions tumours were derived from patients without apparent metastatic spread, we only could evaluate whether there is a trend towards overexpression in relation to tumour progression. Such a trend has been observed for *ATX* and *LO*. It will be most interesting to see in a follow-up study whether expression of these genes will be of prognostic relevance.

Third, RCC are supposed to be immunogenic and are described to express e.g. *RAGE* and certain *MAGE*, genes. In fact, we have described recently the expression of *MAGE-9* as revealed by SSH (Pitzer et al, 1999). Why did we not detect any of these genes in the RCC T9 tissue? First, it should be stated that the RCC T9 does not express *RAGE*, *MAGE-1*, *MAGE-3* and *MAGE-9*. Furthermore, we also know that the serum of the RCC T9 patient does not contain antibodies against a variety of RCC antigens, which have been defined by a SEREX analysis (S. Lubitz et al, unpublished finding). Thus, the RCC T9 apparently is non-immunogenic. These features may explain why we did not detect any immunogenic entities in the T9 RCC even by screening of a testis cDNA array. Besides, it should be noted that SSH would not be the method of choice when searching for immunogenic entities because point mutations, which frequently account for immunogenic tumour antigens, can easily be missed by the suppressive hybridization.

In summary, genes found in this study to be overexpressed in RCC are related to the main features of malignancy, i.e. growth dysregulation, angiogenesis and motility. Furthermore, some of these genes can be considered as central inasmuch as they support survival as well as spreading of tumour cells. This accounts in particular for *EGFR* known to influence proliferation, motility and angiogenesis as well as for *VEGF*, which has bearing on angiogenesis, motility/invasiveness and apoptosis via uPA and survivin/XIAP. Since some of the described molecules are frequently overexpressed in RCC, it will now be of great interest to experimentally support the supposed interconnections and to explore whether these molecules could potentially serve as therapeutic targets.

### ACKNOWLEDGEMENTS

We thank Dr S Matzku, Merck AG, Darmstadt, for helpful suggestions and discussion during preparation of the manuscript. This

 *© 2001 Cancer Research Campaign*

investigation was supported by the Mildred Scheel-Stiftung für Krebshilfe (MZ).

### REFERENCES

Avantagiatti ML (2000) Molecular horizons of cancer therapeutics. *Biochim Biophys Acta* **1470**: 49–59

Bartkova J, Lukas J, Strauss M and Bartek J (1994) The PRAD-1/cyclin D1 oncogene product accumulates aberrantly in a subset of colorectal carcinomas. *Int J Cancer* **58**: 568–573

Bennett BD, Babu-Khan S, Loeloff R, Louis JC, Curran E, Ciltron M and Vassar R (2000) Expression analysis of BACE2 in brain and peripheral tissues. *J Biol Chem* **275**: 20647–20651

Brambilla E, Constantin B, Drabkin H and Roche J (2000) Semaphorin SEMA3F localization in malignant human lung and cell lines: A suggested role in cell adhesion and cell migration. *Am J Pathol* **156**: 939–950

Bremers AJ and Parmiani G (2000) Tumour immunotherapy: the adjuvant treatment of the 21st century? *Crit Rev Oncol Hematol* **34**: 1–25

Brown JM (1999) The hypoxic cell: a target for selective cancer therapy. *Cancer Res* **59**: 5863–5870

Bukowski RM (2000) Cytokine combinations: therapeutic use in patients with advanced renal cell carcinoma. *Semin Oncol* **27**: 204–212

Christensen CR, Klingelhofer J, Tarabykina S, Hulgaard EF, Kramerov D and Lukanidin E (1998) Transcription of a novel mouse semaphorin gene, M-semaH, correlates with the metastatic ability of mouse tumor cell lines. *Cancer Res* **58**: 1238–1244

Conover CA and Powell DR (1991) Insulin-like growth factor (IGF)-binding protein-3 blocks IGF-induced receptor down-regulation and cell desensitization in cultured bovine fibroblasts. *Endocrinology* **129**: 710–716

Conrad PW, Freeman TL, Beitner-Johnson D and Millhorn DE (1999) EPAS1 trans-activation during hypoxia requires p42/p44 MAPK. *J Biol Chem* **274**: 33709–33713

De Plaen E, Lurquin C, Van Pel A, Mariame B, Szikora J, Wölfel T, Sibille C, Chomez P and Boon T (1988) Immunogenic (tum-) variants of mouse tumor P815. Cloning of the gene of tum-antigen P91A and identification of the tum-mutation. *Proc Natl Acad Sci USA* **85**: 2274–2278

Freeman MR, Washecka R and Chung LW (1989) Aberrant expression of epidermal growth factor receptor and HER-2 (erB-2) messenger RNAs in hman renal cancers. *Cancer Res* **49**: 6221–6225

Gerwins P, Skoldenberg E and Claesson-Welsh L (2000) Function of fibroblast growth factors and vascular endothelial growth factors and their receptors in angiogenesis. *Crit Rev Oncol Hematol* **34**: 185–194

Harris SR and Thorgeirsson UP (1998) Tumor angiogenesis: biology and therapeutic prospects. *In Vivo* **12**: 563–570

Hedberg Y, Davoodi E, Roos G, Ljungberg B and Landberg G (1999) Cyclin-D1 expression in human renal cell carcinoma. *Int J Cancer* **84**: 268–272

Hendrix MJ, Seftor EA, Chu YW, Trevor KT and Seftor RE (1996) Role of intermediate filaments in migration, invasion and metastasis. *Cancer Metastasis Rev* **15**: 507–525

Hoffman DM, Gitlitz BJ, Belldegrun A and Figlin RA (2000) Adoptive cellular therapy. *Semin Oncol* **27**: 221–233

Hofmockel G, Tack W and Frohmuller HG (1997) Cyclic interferon alpha treatment in metastatic renal cell carcinoma: results of a phase II study and review of the literature. *Urol Int* **58**: 8–12

Huang YW, Baluna R and Vitetta ES (1997) Adhesion molecules as targets for cancer therapy. *Histol Histopathol* **12**: 467–477

Ishikawa J, Maeda S, Umezu K, Sugiyama T and Kamidono S (1990) Amplification and overexpression of the epidermal growth factor receptor gene in human renal cell carcinoma. *Int J Cancer* **45**: 1018–1021

Jares P, Fernandez PL, Campo E, Nadal A, Bosch F, Aiza G, Nayach I, Traserra J and Cardesa A (1994) PRAD-1/cyclin D1 gene amplification correlates with messenger RNA overexpression and tumor progression in human laryngeal carcinomas. *Cancer Res* **54**: 4813–4817

Kanapuli SP, Singh H, Singh P and Kumar A (1987) Ceruloplasmin gene expression in human cancer cells. *Life Sci* **40**: 2225–2228

Kaplan CD, Morris JR, Wu C and Winston F (2000) Spt5 and spt are associated with active transcription and have characteristics of general elongation factors in D.melanogaster. *Genes Dev* **14**: 2623–2634

Kawagoe H, Stracke ML, Nakamura H and Sano K (1997) Expression and transcriptional regulation of the PD-Ialpha/autotaxin gene in neuroblastoma. *Cancer Res* **57**: 2516–2521

Kerbel RS (2000) Tumor angiogenesis: past, present and the near future. *Carcinogenesis* **21**: 505–515

Khan J, Bittner ML, Chen Y, Meltzer PS and Trent JM (1999) *Biochim Biophys Acta* **1423**: 17–28

Kim I, Moon SO, Koh KN, Kim H, Uhm CS, Kwak HJ, Kim NG and Koh GY (1999) Molecular cloning, expression and characterization of angiopoietin-related protein. *J Biol Chem* **274**: 26523–26528

Kirschmann DA, Seftor EA, Nieva DR, Mariano EA and Hendrix MJ (1999) Differentially expressed genes associated with the metastatic phenotype in breast cancer. *Breast Cancer Res Treat* **55**: 127–136

Kuang WW, Thompson DA, Hoch RV and Weigel RJ (1998) Differential screening and suppression subtractive hybridization identified genes differentially expressed in an estrogen receptor-positive breast carcinoma line. *Nucleic Acid Res* **26**: 1116–1123

Kunkel LM, Monaco AP, Middlesworth W, Ochs HD and Latt SA (1995) Specific cloning of DNA fragments absent from the DNA of a male patient with an X chromosome deletion. *Proc Natl Acad Sci USA* **82**: 4778–4782

Kurzrock R, Ku S and Talpaz M (1995) Abnormalities in the PRAD1 (cyclin d1/bcl-1) oncogene are frequent in cervical and vulvar squamous cell carcinoma lines. *Cancer* **75**: 584–590

Lager DJ, Slagel DD and Palechek PL (1994) The expression of epidermal growth factor receptor and transforming growth factor alpha in renal cell carcinoma. *Mod Pathol* **7**: 544–548

Lamar EE and Palmer E (1984) Y-encoded species-specific DNA in mice: evidence that the Y chromosome exists in two polymorphic forms in inbred strains. *Cell* **37**: 171–177

Lee HY, Murata J, Clair T, Polymeropoulos MH, Torres R, Manrow RE, Liotta LA and Stracke ML (1996) Cloning, chromosomal localization and tissue expression of autotaxin from human teratocarcinoma cells. *Biochem Biophys Res Commun* **218**: 714–719

Lee YH, Tokunaga T, Oshika Y, Suto R, Yanagishawa K, Tomisawa M, Fukada H, Nakano H, Abe S, Tateishi A, Kijima H, Yamazaki H, Tamaoki N, Ueyama Y and Nakamura M (1999) Cell-retained isoforms of vascular endothelial growth factor (VEGF) are correlated with poor prognosis in osteosarcoma. *Eur J Cancer* **35**: 1089–1093

Li YM, Schacher DH, Liu Q, Arkins S, Rebeitz N, McCusker RH Jr, Dantzer R and Kelley KW (1997) Regulation of myeloid growth and differentiation by the insulin-like growth factor I receptor. *Endocrinology* **138**: 362–368

Liang P and Pardee AB (1992) Differential display of eukaryotic messenger RNA by means of polymerase chain reaction. *Science* **257**: 967–971

Lin BT, Brynes RK, Gelb AB, McCourty A, Amin MB and Medeiros LJ (1998) Cyclin D1 expression in renal cell carcinomas and oncocytomas: an immunohistochemical study. *Med Pathol* **11**: 1075–1081

Mark MD, Lohrum M and Püschel AW (1997) Patterning neuronal connections by chemorepulsion: the semaphorins. *Cell Tissue Res* **290**: 299–306

Martin-Satue M and Blanco J (1999) Identification of semaphorin E gene expression in metastatic human lung adenocarcinoma cells by mRNA. *J Surg Oncol* **72**: 18–23

Mickisch GH (1994) Gene therapy on renal-cell carcinoma: magic bullet or tragic insanity? *World J Urol* **12**: 214–223

Meng JY, Kataoka H, Itoh H and Koono M (2001) Amyloid beta protein precursor is involved in the growth of human colon carcinoma cell in vitro and in vivo. *Int J Cancer* **92**: 31–39

Moch H, Schraml P, Bubendorf L, Mirlacher M, Kononen J, Gasser T, Mihatsch MJ, Kallioniemi OP and Sauter G (1999) High-throughput tissue microarray analysis to evaluate genes uncovered by cDNA microarray screening in renal cell carcinoma. *Am J Pathol* **154**: 981–986

Moch H, Gasser T, Amin MB, Torhorst J, Sauter G and Mihatsch MJ (2000) Prognostic utility of the recently recommended histologic classification and revised TNM staging system of renal cell carcinoma: a Swiss experience with 588 tumors. *Cancer* **89**: 604–614

Motzer RJ and Russo P (2000) Systemic therapy for renal cell carcinoma. *J Urol* **163**: 408–417

Nieder C, Niewald M and Schnabel K (1996) Treatment of brain metastases from hypernephroma. *Urol Int* **57**: 17–20

Obata S, Sago H, Mori N, Rochelle JM, Seldin MF, Davidson M, St John T, Taketani S and Suzuki ST (1995) Protocadherin Pcdh2 shows properties similar to, but distinct from those of the classical cadherins. *J Cell Sci* **108**: 3765–3773

Oshika Y, Nakamura M, Tokunaga T, Ozeki Y, Fukushima Y, Hatanaka H, Abe Y, Yamazaki H, Kijima H, Tamaoki N and Ueyama Y (1998) *Int J Oncol* **12**: 541–544

Paul R, Ewing CM, Robinson JC, Marshall FF, Johnson KR, Wheelock MJ and Saac WB (1997) Cadherin-6, a cell adhesion molecule specifically expressed in the proximal renal tubule and renal cell carcinoma. *Cancer Res* **57**: 2741–2748

Pawelec G, Rees RC, Kiessling R, Madrigal A, Dodi A, Baxevanis C, Gambacorti-Passerini C, Masucci G and Zeuthen J (1999) Cells and cytokines in immunotherapy and gene therapy of cancer. *Crit Rev Oncog* **10**: 83–127

*© 2001 Cancer Research Campaign*

Pitzer C, Stassar M and Zöller M (1999) Identification of renal-cell-carcinoma-related cDNA clones by suppression subtractive hybridization. *J Cancer Res Clin Oncol* **125**: 487–492

Raju KS, Alessandri G, Ziche M and Gullino PM (1982) Ceruloplasmin, copper ions and angiogenesis. *J Natl Cancer Inst* **69**: 1183–1188

Ravaud A and Debled M (1999) Present achievements in the medical treatment of metastatic renal cell carcinoma. *Crit Rev Oncol Hematol* **31**: 77–87

Reis M and Faria V (1994) Renal carcinoma: clinical, diagnostic and prognostic aspects. *Arch Esp Urol* **47**: 739–743

Rosen L (2000) Antiangiogenic strategies and agents in clinical trials. *Oncologist* **5** (Suppl 1): 20–27

Sahin U, Türeci Ö, Schmitt H, Cochlovius B, Johannes T, Schmits R, Stenner F, Luo G, Schobert I and Pfreundschuh M (1995) Human neoplasms elicit multiple specific immune responses in the autologous host. *Proc Natl Acad Sci USA* **92**: 11810–11813

Sargent ER, Gomella LG, Belldegrun A, Linehan WM and Kasid A (1989) Epidermal growth factor receptor gene expression in normal human kidney and renal cell carcinoma. *J Urol* **142**: 1364–1368

Shimazui T, Oosterwijk-Wakka J, Akaza H, Bringuir PP, Ruijter E, Debruyne FM, Schalken JA and Oosterwijk E (2000) Alterations in expression of cadherin-6 and E-cadherin during kidney development and in renal cell carcinoma. *Eur Urol* **38**: 331–338

Shimoyama Y, Gotoh M, Terasaki T, Kitajima M and Hirohashi S (1995) Isolation and sequence analysis of human cadherin-6 complementary DNA for the full coding sequence and its expression in human carcinoma cells. *Cancer Res* **55**: 2206–2211

Somers KD and Schechter GL (1992) Genetic alterations in head and neck cancer. *Otolaryngol Clin North Am* **25**: 1065–1071

Stasser M, Pitzer C, Zoller M (1999) Down-regulation of TNF receptor-associated protein–2/p 97 in renal cell carcinoma. *Oncology Res* **11**: 85–90

Stracke ML, Krutzsch HC, Unsworth EJ, Arestad A, Cioce V, Schiffmann E and Liotta LA (1992) Identification, purification and partial sequence analysis of autotaxin, a novel motility-stimulating protein. *J Biol Chem* **267**: 2524–2529

Stracke ML, Clair T and Liotta LA (1997) Autotaxin, tumor motility-stimulating exophosphodiesterase. *Adv Enzyme Regul* **37**: 135–144

Thompson EW, Yu M, Bueno J, Jin L, Maiti SN, Palao-Marco FL, Pulyaeva H, Tamborlane JW, Tirgari R and Wapnir I (1994) Collagen induced MMP-2 activation in human breast cancer. *Breast Cancer Treat* **31**: 357–370

Tian H, McKnight SL and Russell DW (1997) Endothelial PAS domain protein 1 (EPAS1), a transcription factor selectively expressed in endothelial cells. *Genes Dev* **11**: 72–82

Tomisawa M, Tokunaga T, Oshika Y, Tsuchida T, Fukushima Y, Sato H, Kijima H, Yamazaki H, Ueyama Y, Tamaoki N and Nakamura M (1999) Expression pattern of vascular endothelial growth factor isoform is closely correlated with tumor stage and vascularisation in renal cell carcinoma. *Eur J Cancer* **35**: 133–137

Tran J, Rak J, Sheehan C, Saibil SD, LaCasse E, Korneluk RG and Kerbel RS (1999) Marked induction of the IAP family antiapoptotic proteins survivin and XIAP by VEGF in vascular endothelial cells. *Biochem Biophys Res Commun* **264**: 781–788

Ullrich A and Schlessinger J (1990) Signal transduction by receptors with tyrosine kinase activity. *Cell* **61**: 203–212

Vassar R, Bennett BD, Babu-Khan S, Kahn S, Mendiaz EA, Denis P, Teplow DB, Ross S, Amarante P, Loeloff R, Luo Y, Fisher S, Fuller J, Edenson S, Lile J, Jarosinski MA, Biere AL, Curran E, Burgess T, Louis JC, Collins F, Treanor J, Rogers G and Citron M (1999) β-secretase cleavage of Alzheimer's amyloid precursor protein by the transmembrane aspartic protease BACE. *Science* **286**: 735–741

Vaupel PW (1997) The influence of tumor blood flow and microenvironmental factors on the efficacy of radiation, drugs and localized hyperthermia. *Klin Padiatr* **209**: 243–249

Wang RF and Rosenberg SA (1999) Human tumor antigens for cancer vaccine development. *Immunol Rev* **170**: 85–100

Weidner N (1995) Intratumor microvessel density as a prognostic factor in cancer. *Am J Pathol* **147**: 9–19

Weinberg RA (1995) The retinoblastoma protein and cell cycle control. *Cell* **81**: 323–330

Weiss H, Friedrich T, Hofhaus G and Preis D (1991) The respiratory chain NADH dehydrogenase (complex I) of mitochondria. *Eur J Biochem* **196**: 563–576

Wen Y and Shatkin AJ (1999) Transcription elongation factor hSPT5 stimulates mRNA capping. *Genes Dev* **13**: 1774–1779

Wiesener MS, Turley H, Allen WE, William C, Eckardt KU, Talks KL, Wood SM, Gatter KC, Harris AL, Pugh CW, Ratcliffe PJ and Maxwell PH (1997) Induction of endothelial PAS domain protein-1 by hypoxia: characterization and comparison with hypoxia-inducible factor-1 alpha. *Blood* **92**: 2260–2268

Williamson JR, Chang K, Rowold E, Marvel J, Tomlinson M, Sherman WR, Ackermann KE and Kilo C (1985) Sorninil prevents diabetes-induced increases in vascular permeability but does not after collagen-cross-linking. *Diabetes* **37**: 703–705

Xin H, Stephans JC, Duan X, Harrowe G, Kim E, Grieshammer U, Kingsley C and Giese K (2000) Identification of a novel aspartic-like protease differentially expressed in human breast cancer cell lines. *Biochim Biophys Acta* **1501**: 125–137

Yamaguchi Y, Narita T, Inukai N, Wada T and Handa H (2001) Spt genes: key players in the regulation of transcription, chromatin structure and other cellular processes. *J Biochem* **129**: 185–191

Yang Y, Mou L, Liu N and Tsao MS (1999) Autotaxin expression in non-small-cell lung cancer. *Am J Respir Cell Mol Biol* **21**: 216–222

Yoshida K, Tosaka A, Takeuchi S and Kobayashi N (1994) Epidermal growth factor receptor content in human renal cell carcinomas. *Cancer* **73**: 1913–1918

Yu AE, Hewitt RE, Conner EW and Stetler-Stevenson WG (1997) Matrix metalloproteinases. Novel targets for directed cancer therapy. *Drugs Aging* **11**: 229–244

Zhang SY, Caamano J, Cooper F, Guo X and Klein-Szanto AJ (1994) Immunohistochemistry of cyclin D1 in human breast cancer. *Am J Clin Pathol* **102**: 695–698

*© 2001 Cancer Research Campaign*
